# Laser-Modified Ti Surface Improves Paracrine Osteogenesis by Modulating the Expression of DKK1 in Osteoblasts

**DOI:** 10.3390/jfb14040224

**Published:** 2023-04-16

**Authors:** Jorge Felipe Lima Teixeira, João Antônio Chaves de Souza, Fernando Augusto Cintra Magalhães, Guilherme José Pimentel Lopes de Oliveira, José Bernardo de Santis, Carlos Alberto de Souza Costa, Pedro Paulo Chaves de Souza

**Affiliations:** 1 Department of Physiology and Pathology, School of Dentistry, São Paulo State University, Araraquara 14801-385, Brazil; 2 School of Dentistry, Federal University of Goiás, Goiânia 74605-020, Brazil; 3 Nursing Department, Federal University of Maranhão, Imperatriz 65905-240, Brazil; 4 Department of Periodontology and Implant Dentistry, School of Dentistry, Federal University of Uberlândia, Uberlândia 38405-320, Brazil; 5 Department of Basic and Oral Biology, Bone Research Lab, School of Dentistry of Ribeirão Preto, University of São Paulo, Ribeirão Preto 14040-904, Brazil; 6 Innovation in Biomaterials Laboratory (iBioM), School of Dentistry, Federal University of Goiás, Goiânia 74605-020, Brazil

**Keywords:** laser ablation, Ti, nanotopography, osteoblast, osseointegration

## Abstract

Titanium surface modifications are widely used to modulate cellular behavior by recognition of topographical cues. However, how those modifications affect the expression of mediators that will influence neighboring cells is still elusive. This study aimed to evaluate the effects of conditioned media from osteoblasts cultured on laser-modified titanium surfaces on the differentiation of bone marrow cells in a paracrine manner and to analyze the expression of Wnt pathway inhibitors. Mice calvarial osteoblasts were seeded on polished (P) and Yb:YAG laser-irradiated (L) Ti surfaces. Osteoblast culture media were collected and filtered on alternate days to stimulate mice BMCs. Resazurin assay was performed every other day for 20 days to check BMC viability and proliferation. After 7 and 14 days of BMCs maintained with osteoblasts P and L-conditioned media, alkaline phosphatase activity, Alizarin Red staining, and RT-qPCR were performed. ELISA of conditioned media was conducted to investigate the expression of Wnt inhibitors Dickkopf-1 (DKK1) and Sclerostin (SOST). BMCs showed increased mineralized nodule formation and alkaline phosphatase activity. The L-conditioned media enhanced the BMC mRNA expression of bone-related markers *Bglap*, *Alpl*, and *Sp7*. L-conditioned media decreased the expression of DKK1 compared with P-conditioned media. The contact of osteoblasts with Yb:YAG laser-modified Ti surfaces induces the regulation of the expression of mediators that affect the osteoblastic differentiation of neighboring cells. DKK1 is among these regulated mediators.

## 1. Introduction

Osseointegrated implants are widely used with high clinical success rates and predictability in oral rehabilitation [1,2]. Since the mid-1960s, titanium (Ti) has been considered a reliable material for bone implantations due to its excellent biocompatibility, corrosion resistance, and mechanical properties, providing stable implant anchorage in most cases [3,4,5].

The clinical success of a dental implant depends on many factors, including surgical procedure, bone quality, and how fast and uniform the osseointegration occurs [6,7]. Faster osseointegration allows an earlier loading and increases success rates even in patients with poor bone quality or systemic conditions [8].

Disordered microstructures are common cues that influence cell morphology and behavior [9,10,11]. Thus, the specialized industry widely investigates Ti surface modification protocols and alternatives to increase the quality of dental implant osseointegration [12]. These modifications usually involve chemical treatment or particle incrustation, raising the product’s final cost and adding potential contaminants to its surface [13,14].

High-power laser irradiation is a cheaper, faster, and reproducible alternative for Ti surface modifications that can eliminate chemicals and residual particles adhered to the substrate. This method creates different roughness and topographical patterns with a superficial oxide layer of nanometer thickness that improves corrosion resistance and biocompatibility and plays a critical role in osseointegration [15,16,17,18]. Laser-irradiated Ti surfaces exhibit high wettability, increased cell spreading, and adhesion to the material [10,19,20]. Previous in vivo studies have demonstrated that high-power laser Ti modifications produced nano- and microstructures capable of accelerating the implant osseointegration [16,18], which is a complex process that includes the recruitment and differentiation of osteoblasts, mainly controlled by the early maturation runt-related transcription factor 2 (Runx2) and the downstream transcriptional regulator Osterix (Sp7). Differentiated osteoblasts secrete bone matrix proteins, including collagen type 1 alpha 1 (Col1a1), osteocalcin (OC), and alkaline phosphatase (Alp), that will regulate mineralized matrix deposition and new bone formation [21,22].

For effective implant integration, osteogenesis must occur in two different sites: in contact with the Ti implant surface and at a distance, on the host bone surface [3,23]. The distance osteogenesis will connect the host bone to the implant surface, while the contact osteogenesis will enable an effective bone apposition. Although contact osteogenesis seems to require other factors to be triggered, histological evidence demonstrates that implant surface modifications play a critical role in paracrine osteogenesis and might accelerate the final implant osseointegration [23,24,25].

To date, in vitro studies using the contact osteogenesis approach showed that modifications on the Ti surface can accelerate and improve implant/bone interface consolidation [5,10,21]. It has been demonstrated that micro- and nanopatterns on surface topography influence cell morphology, increasing the chemotactic signal of cell adhesion and affecting integrin receptors and the subsequent secretion of cytokines that may modulate an osteogenic differentiation [26]. Our group has previously shown that irradiation of Ti with high-power laser creates a surface that can induce osteoblastic differentiation, increasing the mRNA expression of osteoblast transcription factors (*Runx2* and *Sp7*), matrix proteins (*Col1a1*, *Spp1*, and *Bglap*), and enzymes (*Alpl*) [10]. However, whether and how this microenvironment may influence bone formation in a paracrine manner has yet to be investigated. Understanding how Ti surface modifications affect the expression of mediators of osteoblastic differentiation that may act in a paracrine manner will shed a new light on the comprehension of the mechanisms of osseointegration.

The Wnt/β-catenin signaling pathway is essential to embryonic skeleton development; it modulates bone formation and resorption by promoting osteoblast and osteocyte differentiation, maturation, and survival and inhibiting osteoclastogenesis [27]. Its activation by binding Wnt proteins to a Frizzled (Fzd) receptor and co-receptor (LDL receptor-related protein (Lrp5 or Lpr6)) results in the accumulation of non-phosphorylated β-catenin in the cytoplasm that will eventually be translocated into the nucleus and regulates gene transcription [28]. The Dickkopf (Dkk) family is a well-known group of proteins that modulate the Wnt signaling pathway; they prevent the formation of the Wnt–Fzd–Lrp complex binding to co-receptor Lpr5/6 [29]. Even though Wnt proteins and their downstream signaling components have been widely studied and targeted as bone formation modulators on modified surfaces [30,31], the role of Wnt signaling inhibitors on distance bone formation within the implant/bone interface microenvironment remains unclear.

Here, we report that conditioned culture media from osteoblasts cultured on laser-modified Ti surface stimulates bone marrow cell (BMC) osteoblastic differentiation in a paracrine manner. A possible mechanism is the decrease in the expression of DKK1 by osteoblasts cultured on this surface.

## 2. Materials and Methods

The in vitro study was approved by the local Ethical Committee for Animal Experimentation from the Faculty of Dentistry of Araraquara (protocol # 23/2013, approved on March 2016) and conducted according to the ARRIVE guidelines. The experiments were carried out in triplicate, with three independent experiments.

### 2.1. Ti Disks Preparation and Characterization

Commercially pure (grade 4) Ti disks (175 mm^2^ of area and 2 mm thickness) were sequentially polished under water-cooling with a 320- to 1200-grit grinding paper. They were immersed in acetone PA ACS (Qhemis, Indaiatuba, SP, Brazil) and ultrasonically cleaned for 15 min and washed with deionized water and 100% ethanol (Qhemis, Indaiatuba, SP, Brazil). The disks were finally rinsed with distilled water for 15 min and dried for 90 min in an incubator at 60 °C (Orion 515, Fanem, São Paulo, SP, Brazil). The polished samples were divided into two experimental groups, polished Ti (P Ti) and laser Ti (L Ti). The laser surface was obtained by laser ablation with a pulsed Yb:YAG laser (Omnimark 20F, Ominitek Tecnologia Ltda, São Paulo, Brazil) as previously described [10]. Briefly, the surfaces were irradiated in an ambient atmosphere, and the laser beam was perpendicular to the surface with a fixed focal length of 170 mm. The laser wavelength was 1064 nm, scanning speed 100 mm/s, pulse duration 100 ns, average pulse power 10 kW, spot diameter 50 μm and cutoff distance 0.1 mm.

To access the surface topography, the specimens were characterized by scanning electron microscopy (SEM) (JMS-T33A, JEOL, Tokyo, Japan). The average surface roughness measured in our previous publication [10] was 0.3151 ± 0.0069 μm for the P surface and 10.57 μm ± 0.39 for the L surface.

### 2.2. Isolation and Culture of Mouse Calvarial Osteoblasts

Primary osteoblasts were isolated from neonatal calvarial bones obtained from 2- to 3-day-old mice (Mus musculus C57Bl/6) using sequential enzymatic digestion [32]. The cells obtained were seeded in plain minimum essential medium α (α-MEM, Invitrogen, Carlsbad, CA, USA) supplemented with penicillin/streptomycin (Pen-Strep, Sigma Aldrich, St. Louis, MO, USA) and 10% fetal bovine serum (FBS, Sigma Aldrich, St. Louis, MO, USA). After three days of incubation, 20,000 cells were seeded on Ti disks with L or P surface (24-well plates) in osteogenic differentiation media and α-MEM-supplemented with 10 mmol/L β-glycerophosphate and 2 mmol/L ascorbic acid (Sigma Aldrich, Carlsbad, CA, USA). The OM was collected and filtered every other day to stimulate the bone marrow cell culture and analyze bone metabolism, as described below.

### 2.3. Isolation and Culture of Mouse Bone Marrow Cells

BMCs were isolated from the tibia and femur of 4-week-old mice as previously described [33]. A total of 5 × 10^5^ cells were seeded in 96-well plates in complete α-MEM. After 24 h, the complete medium was replaced for the OM medium obtained from calvarial osteoblast-like cultures seeded over P or L Ti surfaces; every 48 h, the fresh conditioned medium was collected from calvarial osteoblasts cultured in parallel to the BMC cultures, filtered and used to stimulate BMCs.

### 2.4. Alamar Blue

Resazurin (Alamar Blue, Sigma Aldrich, St. Louis, MO, USA) assay was performed every other day after the first day of culture for 20 days, according to the manufacturer’s protocol, to analyze the BMC’s viability and proliferation. The 1:10 Alamar Blue solution was directly added into the culture wells, and the plates were incubated for 1 h at 37 °C. Cell viability was assessed by the proportional absorbance at 570 nm in a microplate reader (Power Wave XS, BioTek Instruments, Winooski, VT, USA).

### 2.5. Alkaline Phosphatase Activity

After 7 and 14 days, the osteoblasts were lysed using 500 µL of a 50 mmol/L Tris–HCl solution (pH 7.4) containing 0.5% of Nonidet P40. The total protein content was quantified using a bicinchoninic acid assay (BCA Protein Assay Kit, Thermo Scientific, Rockford, IL, USA). ALPase activity was assessed by measuring the release of thymolphthalein from thymolphthalein monophosphate using a commercial test kit (Labtest Diagnostica SA, Belo Horizonte MG, Brazil). According to the estimated protein amount, 20 µg of total protein was added to a solution of thymolphthalein monophosphate (22 mmol/L) and diethanolamine buffer solution (0.3 mmol/mL, pH 10.1) and incubated for 40 min at 37 °C. About 200 µL of a Na_2_CO_3_ (0.09 mmol/mL) and NaOH (0.25 mmol/mL) solution was added and incubated at 37 °C for additional 10 min; the color gradient was measured by the absorbance at 590 nm in a microplate reader (Power Wave XS, BioTek Instruments, USA). ALPase activity was calculated from a standard curve using alkaline phosphatase as the standard in a range varying from 22.5 to 900 μU.

### 2.6. Alizarin Red Staining

The mineral deposition of osteoblast-like cells treated with OM was accessed by Alizarin Red staining at 14 and 21 days after the cells were cultured in 96-well plates. The cells were rinsed twice with PBS at 37 °C and then fixed with 150 µL of 4% paraformaldehyde for 10 min. After rinsing the cells again with PBS, 50 µL of 10% *w*/*v* Alizarin Red (Sigma Aldrich, St. Louis, MO, USA) staining solution was added to each well at room temperature for 10 min, the staining solution was removed, and the wells were rinsed twice with PBS. The mineralization was quantified by dissolving stained nodules with 200 µL of 10% *w*/*v* cetylpyridinium chloride (Sigma Aldrich, St. Louis, MO, USA) solution. The dissolved nodules were transferred to 96-well plates and read at 562 nm in a microplate reader (Power Wave XS, BioTek Instruments, USA).

### 2.7. Real-Time PCR

Total RNA was extracted from seeded BMCs treated with OM for 14 days using an affinity column method according to the manufacturer’s protocol (RNAqueous-micro Kit, Thermo Scientific, Rockford, IL, USA). The RNA from each well was quantified by UV absorbance (Eppendorf, Hamburg, Germany) and considered acceptable when the 260/280 nm absorbance ratio was above 1.8. Three hundred nanograms of total RNA was converted into cDNA using random hexamer primers and reverse transcriptase in a total reaction volume of 20 µL (high-capacity cDNA synthesis kit, Applied Biosystems).

Quantitative real-time PCRs (qPCRs) were performed to assess the expression levels of the genes encoding the transcription factors *Runx2* (forward primer, 5′-CCTGAACTCTGCACCAAGTCCT-3′; reverse primer, 5′-TCATCTGGCTCAGATAGGAGGG-3′) and Osterix (*Sp7*) (forward primer, 5′-TGCTTGAGGAGGAAGTTCAC-3′; reverse primer, 5′-AGGTCACTGCCCACAGAGTA-3′), and bone-specific proteins osteopontin/secreted phosphoprotein (*Spp1*) (forward primer, 5′-TCACCATTCGGATGAGTCTG-3′; reverse primer, 5′-ACTTGTGGCTCTGATGTTCC-3′), alkaline phosphatase (*Alpl*), α1 chain of collagen type 1 (*Col1a1*) (forward primer, 5′-CCTCAGGGTATTGCTGGACAAC-3′; reverse primer, 5′-CAGAAGGACCTTGTTTGCCAGG-3′) and osteocalcin/bone gamma-carboxy glutamic acid protein (*Bglap*) (forward primer, 5′-GCAATAAGGTAGTGAACAGACTCC-3′; reverse primer, 5′-CCATAGATGCGTTTGTAGGCGG-3′). The 20 µL total volume reactions included a TaqMan qPCR master mix (Applied Biosystems), cDNA, deionized water, and species-specific predesigned and optimized pairs of primers and probes (TaqMan gene expression assays).

The relative levels of gene expression were determined using the quantification cycle by the standard curve method using the gene encoding to β-actin (*Actb*) as the reference gene. Results were expressed as fold change over the levels of expression of the normalized target gene determined in cDNA prepared from the P Ti sample group.

### 2.8. ELISA

The ELISA tests for Dkk-1 (# MKK100) and Sclerostin (# MSST00) were performed following the manufacturer’s recommended protocol (R&D Systems, Minneapolis, MN, USA). For these assays, aliquots of the calvarial osteoblast culture supernatants (OM) were collected at 0, 2, 4, 6, 8, 10, and 12 days and were mixed to obtain pooled samples. Six different pools were evaluated per group (n = 6).

### 2.9. Statistics

Data from Alamar Blue, RT-qPCR, alkaline phosphatase activity, Alizarin red, and ELISA assays were submitted to t-tests followed by Welch’s correction since they adhered to the normal distribution (Shapiro–Wilk, *p* > 0.05). The level of significance was 95% (*p* < 0.05). Results were represented as mean + SD.

## 3. Results

### 3.1. Ti Surface Characterization

**Figure 1:**
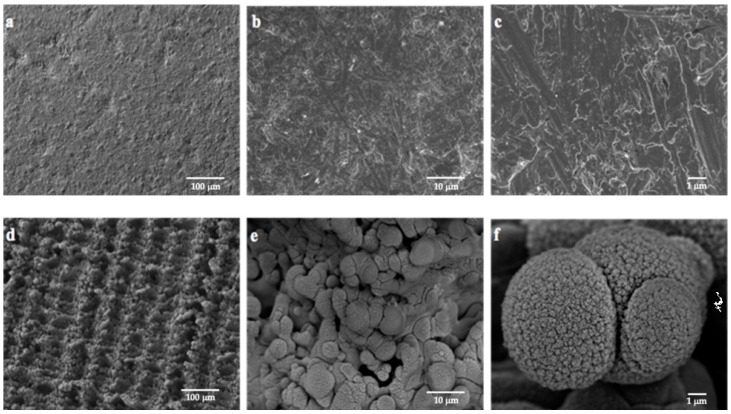
Representative SEM images of polished Ti surface in ×200 (**a**), ×2000 (**b**), and ×10,000 (**c**); and laser Ti surface in ×200 (**d**), ×2000 (**e**), and ×10,000 (**f**).

The results of the SEM analysis revealed topographic differences between the P and L Ti surfaces. The polished surface was homogeneous with minor residual roughness from the manufacturing process (Figure 1a–c). In contrast, laser surfaces generally presented an irregular surface, characterized by the presence of spheres and protrusions of different diameters due to the irradiation pattern (Figure 1d–f). Longitudinal channel-shaped depressions with different depths and widths were observed. At high magnification, it was possible to detect in the L surface the presence of secondary nano-roughness inside the spheres caused by the Ti ablation. Therefore, laser irradiation created a nano-to-micro hybrid surface (Figure 1f).

### 3.2. Cell Viability and Proliferation

**Figure 2:**
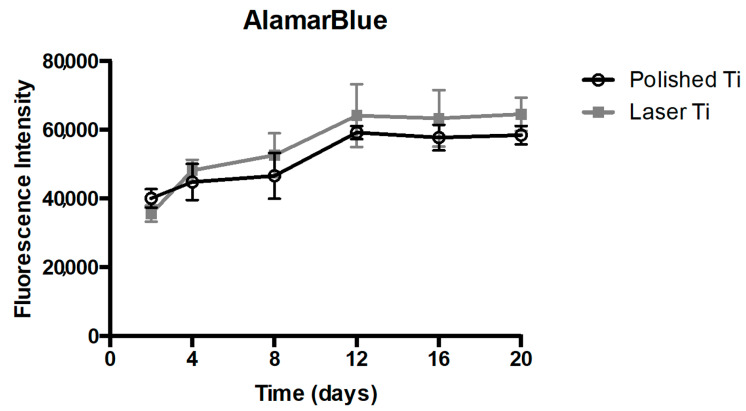
BMC proliferation and viability assessed by Alamar Blue assay. Mean (±SD) of fluorescence intensity at 2, 4, 8, 12, 16, and 20 days (n = 8) (*p* > 0.05).

The effect of surface topography on the osteoblastic expression of proliferation/differentiation factors was first investigated on the ability of the osteoblastic conditioned medium to induce BMC viability and proliferation. The cells were treated with the conditioned medium from osteoblasts cultured in P and L Ti surfaces. For all the experimentation periods, no significant difference between the groups was observed (Figure 2) (*p* > 0.05).

### 3.3. Conditioned Medium from Osteoblasts Cultured on Laser-Modified Surface Affects Osteoblastic Genes Expression in BMCs

**Figure 3:**
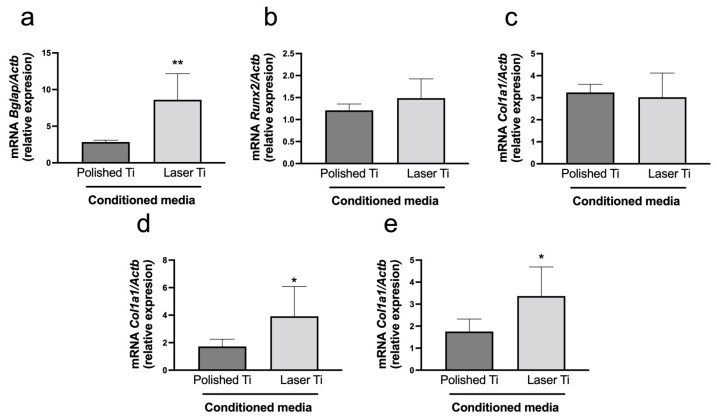
mRNA relative expression of osteoblast phenotypical marks after 14 days of culture of BMCs treated with P and L Ti surface-conditioned media. Mean (±SD) of mRNA expression of *Bglap* (**a**), *Runx2* (**b**), *Col1a1* (**c**), *Alpl* (**d**), and *Sp7* (**e**), (n = 8). Real-time PCR (* *p* < 0.05; ** *p* < 0.01).

The effects of the laser-modified Ti surface on the osteoblast production of differentiation factors were then investigated on the expression of mRNAs, encoding for transcription factors (*Runx2* and *Sp7*) and osteoblast phenotypic markers (*Alpl*, *Col1a1*, and *Bglap*), by BMCs treated with the conditioned medium obtained from osteoblasts cultured on P and L surfaces. After 14 days, the L surface-conditioned medium upregulated the mRNA expression of the transcription factor *Sp7* and the phenotypic markers *Bglap* and *Alpl* (Figure 3).

### 3.4. Laser Ti Surface Stimulates ALPase Activity in BMC

**Figure 4:**
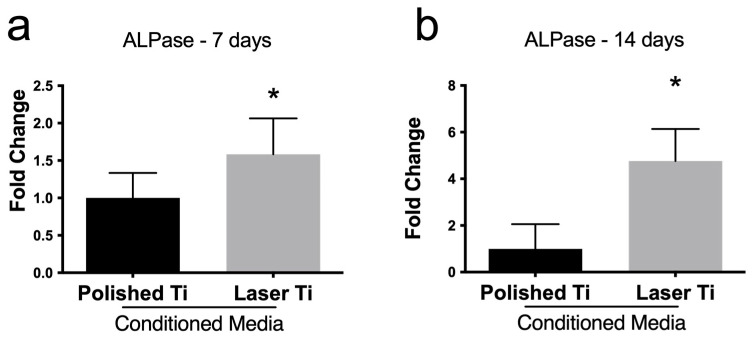
ALPase activity of BMCs treated with P and L Ti surface-conditioned medium at the 7th and 14th day of culture. Mean (±SD) of spectrophotometric quantification of ALPase activity in BMC cultures at (**a**) 7 and (**b**) 14 days (n = 8) (* *p* < 0.05).

After 7 and 14 days, the activity of ALPase was increased in BMCs treated with the conditioned medium obtained from the culture of osteoblasts grown on L Ti-irradiated surfaces, compared with BMCs treated with the medium obtained from osteoblasts grown on the P surfaces (Figure 4).

### 3.5. Laser Ti Surface Stimulates Mineralized Nodule Formation

**Figure 5:**
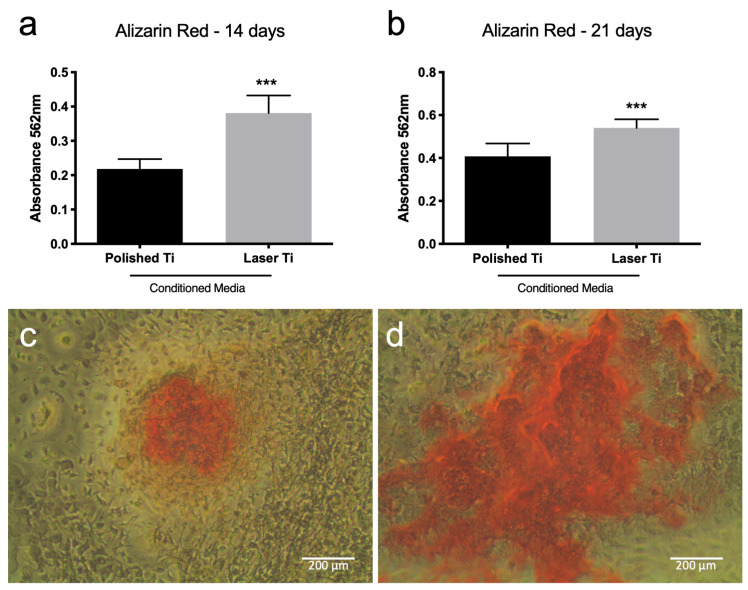
Mineralized matrix deposition of BMCs maintained in P or L Ti-conditioned medium after 14 (**a**) and 21 (**b**) days of culture. Mean (±SD) of absorbance after mineralized nodule dissolution (n = 8) (*** *p* < 0.001). Representative images of the nodules formed by BMCs cultured on P-conditioned media (**c**) or L-conditioned media (**d**) at 21 days.

The formation of a mineralized extracellular matrix is what defines fully differentiated and active osteoblasts. To investigate how the conditioned medium from osteoblasts cultured on different Ti surfaces affected BMC differentiation on fully differentiated osteoblasts, we quantified the calcified nodules formed in BMC cultures treated with a P or L Ti surface-conditioned medium after 14 and 21 days of culture. Active formation of mineralized nodules was observed in both groups and experimental periods. After 14- and 21-day cultures, BMCs treated with the laser Ti surface-conditioned medium showed higher calcified matrix formation compared with cells treated with the polished Ti surface-conditioned medium (Figure 5, Supplemental Figure S1) (*p* < 0.05).

### 3.6. Laser Ti Surface Downregulates Wnt Signaling Inhibitor in Osteoblasts

**Figure 6:**
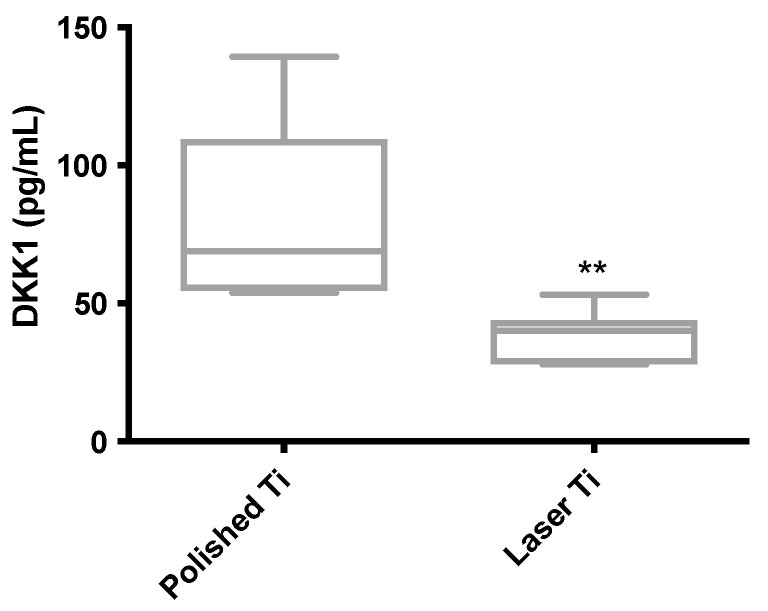
Dkk-1 protein measured by ELISA is downregulated in laser Ti surface samples. Dkk-1 was measured by ELISA in osteoblasts cultured on P and L Ti surfaces. Mean (±SD) expression of Dkk-1, OPN, and TGF-beta proteins (n = 6) (** *p* < 0.01).

To investigate the impact of the laser-modified surface on the expression of Wnt signaling inhibitors, we performed an ELISA for Dkk-1 and Sclerostin using the supernatant of osteoblasts cultured on P and L surfaces. The expression of Dkk-1 was decreased in the L surface group compared with that in the polished P surface group (Figure 6). The protein Sclerostin was not detected in all groups.

## 4. Discussion

The implant osseointegration is environment-dependent and largely influenced by Ti surface topographical properties [5,34]. Several techniques aiming for micro- and nanostructured surface modifications have been proposed to modulate this biological process [35]. Laser surface treatments have often been described as a highly controllable approach to improving the cellular response to the implant surface due to the creation of micro- and nanometric features [16,18]. Our group has previously fabricated and characterized a nano-to-micro roughened surface produced by Yb:YAG laser ablation [10]. Besides changing the surface roughness, the melting caused by laser ablation led to the incorporation of atmospheric gases and the formation of a TiO_2_ layer containing both the rutile and anatase phases [10]. In agreement with previous publications showing that the anatase phase of titanium plays an important role in cell adhesion, proliferation, and differentiation [36,37], our surface increased the expression of osteoblastic genes and their differentiation, as shown by the increased ALPase activity and mineral nodule deposition [10]. Besides the well-documented direct effect on bone cells in contact with the implant surface, laser ablation might influence cytokine and growth factor expression and release patterns that dictate paracrine osteogenesis in the bone/implant microenvironment [38,39,40]. Distance osteogenesis occurs on the host bone at the peri-implant area, and it is an important event for complete osseointegration and endosseous healing [3,23]. Here, we report that the laser-ablated surface decreased the expression of Wnt modulator Dkk-1 in osteoblast cultures. The conditioned medium obtained from these cultures induced the osteogenic differentiation and activity of BMCs in a paracrine fashion.

Although the laser Ti surface-conditioned medium did not affect cell proliferation and viability, it enhanced BMC ALPase activity and mineralized nodule formation compared with the P-conditioned medium group, suggesting that the ALPase activity of bone marrow stromal cells present in the host bone plays an important role in the bone matrix calcification of the peri-implant area. It has been demonstrated that the Ti-conditioned medium enhanced pre-osteoblast adhesion and activated key signaling proteins related to bone metabolism [38]. The laser surface-conditioned medium also increased the mRNA expression of *Sp7*, *Bglap*, and *Alpl*. It was recently reported that when stimulated with a conditioned medium obtained from RAW cells cultured on microstructured Ti topographies, bone marrow stem cells exhibited nearly 1.5-fold higher expression of osteogenic genes and production of mineralized nodules compared with the direct stimulation of Ti surfaces [40]. Recently, laser-induced micro–nano patterned structures were proven to overlay isotropic and anisotropic cues that could influence cell shape and induce nuclear orientation and activation of the Wnt/β-catenin pathway, signaling to increase the expression and activation of integrin α5, integrin β1, cadherin 2, Runx2, OPN, and OCN [30]. The decreased expression of the Wnt/β-catenin inhibitor Dkk-1, allied to the subsequent increased release of bone-related proteins induced by laser-irradiated Ti surface, may explain the modulation of BMC differentiation.

The Wnt/β-catenin signaling pathway is an important mechanism that interferes in cell proliferation, cell polarity, and cell fate determination during embryonic and postnatal development [24]. Bone diseases, such as osteoporosis, pseudoglioma syndrome, sclerosteosis, and van Buchem’s disease, have been associated with atypical Wnt signaling [41]. The Wnt signaling pathway is one of the most critical regulators of bone tissue formation and homeostasis. The Wnt/β-catenin inhibitor Dkk-1 negatively regulates bone mass, and the reduction of this molecule results in increased bone mass in mice [42]. In the present study, the expression of Dkk-1 decreased on osteoblasts cultured on the L surface group, suggesting that besides the direct mechanical stimulation on cellular cytoskeleton and its integrin receptors [43], the laser treatment might modulate osteogenesis occurring within the implant/bone microenvironment by downregulating the Wnt canonical signaling inhibitor. We also analyzed the expression of another important inhibitor of Wnt signaling, sclerostin, an important factor regulating bone mass [44]. However, sclerostin (SOST) mRNA was not detected in any experimental groups. Since osteocytes are the leading producers of SOST, several growth factors and bone-related proteins have been deposited in ECM during mineralization [45,46]. We believe that the levels of SOST present in the osteoblast culture supernatant were insufficient to be reactive in ELISA measurement.

As demonstrated, the laser-modified Ti surface can affect bone cells at a distance, enhancing paracrine mineralized tissue formation by downregulating Dkk-1 and modulating osteoblastic lineage transcription factors and key protein expression. Additionally, the conditioned medium is a worthy tool for stimulating bone regeneration. Cultured MSCs secrete several growth factors and cytokines into the medium with the potential to stimulate tissue regeneration [47]. Pioneering studies have reported that the conditioned medium from mesenchymal stem cells improved bone regeneration in critical-sized calvarial defects in rats [48]. Moreover, the bone-conditioned medium enhanced osteoblastic differentiation on collagen membranes [49] and inorganic bovine bone grafts [50]. Thus, understanding how cells interact with different surfaces will provide new avenues for bone engineering. There are a plethora of surface modifications nowadays [51], and they can be used not only to accelerate the osseointegration of implanted devices but also to serve as bioreactors for the production of “bone growth media”. However, further molecular investigations, gene editing tools, and in vivo studies are still needed to extend the understanding of the underlying mechanisms governing surface-induced paracrine osteogenesis.

## 5. Conclusions

Considering the methodology used in the present study, one may conclude that modifying the Ti surface with Yb:YAG laser positively impacts osteoblast behavior, enhancing the expression of key osteoblastic markers and downregulating the Wnt signaling inhibitor Dkk-1. The pre-conditioned medium stimulated the bone marrow cells into an osteogenic phenotype showing that laser-ablated surfaces might affect the bone tissue regeneration process occurring at a distance in the bone/implant microenvironment.

## Data Availability

Not applicable.

## Supplementary Materials

The following supporting information can be downloaded at: https://www.mdpi.com/article/10.3390/jfb14040224/s1, Figure S1: Representative images of BMCs cultured in P-conditioned or L-conditioned media for 7 and 21 days.
