# Supplementary material for: Laser-Modified Ti Surface Improves Paracrine Osteogenesis by Modulating the Expression of DKK1 in Osteoblasts
Source: J Funct Biomater. 2023 Apr 16;14(4):224. doi: 10.3390/jfb14040224 (PMC10145280; doi:10.3390/jfb14040224)
Supplement: Supplementary file 1 [file jfb-14-00224-s001.zip › jfb-2239793-supplementary.pdf]

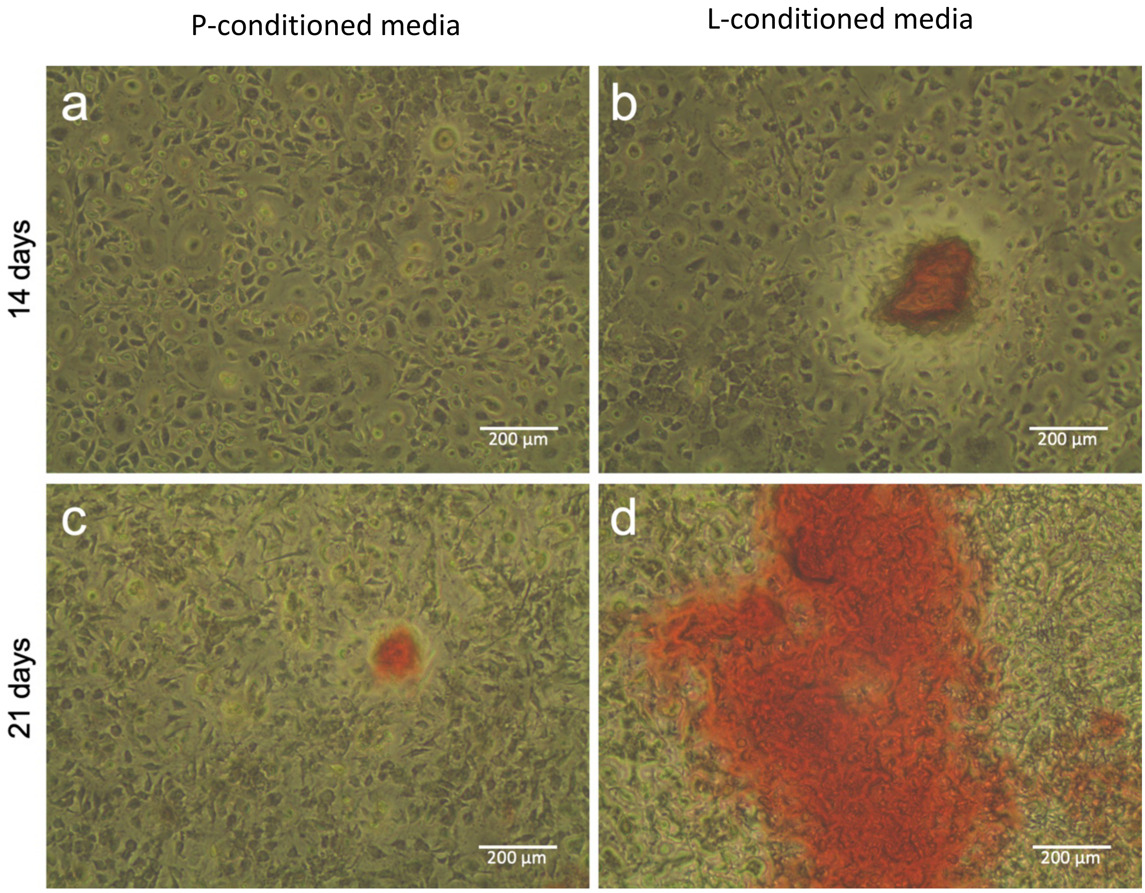

Supplemental Figure S1. Representative images of BMCs cultured in P-conditioned or L-conditioned media for 7 and 21 days.
